# Immune Checkpoint Inhibitors in Cancer Therapy

**DOI:** 10.3390/curroncol29050247

**Published:** 2022-04-24

**Authors:** Yavar Shiravand, Faezeh Khodadadi, Seyyed Mohammad Amin Kashani, Seyed Reza Hosseini-Fard, Shadi Hosseini, Habib Sadeghirad, Rahul Ladwa, Ken O’Byrne, Arutha Kulasinghe

**Affiliations:** 1 Department of Molecular Medicine and Medical Biotechnology, University of Naples Federico II, 80138 Naples, Italy; shiravandy@gmail.com; 2 Faculty of Pharmaceutical Sciences, PES College of Pharmacy, PES University, Bengaluru 560085, India; selmakhodadadi@gmail.com; 3 Student Research Committee, Shiraz University of Medical Sciences, Shiraz 7134814336, Iran; kashanimohammadamin@gmail.com; 4 Department of Biochemistry, School of Medicine, Tehran University of Medical Sciences, Tehran 1417613151, Iran; sr.hoseinifard@gmail.com; 5 Cellular and Molecular Research Center, Research Institute for Health Development, Kurdistan University of Medical Sciences, Sanandaj 6617713446, Iran; shadihosseini7@gmail.com; 6 The University of Queensland Diamantina Institute, Faculty of Medicine, The University of Queensland, Brisbane, QLD 4102, Australia; h.sadeghirad@uq.net.au; 7 Princess Alexandra Hospital, Brisbane, QLD 4102, Australia; rahul.ladwa@health.qld.gov.au (R.L.); k.obyrne@qut.edu.au (K.O.)

**Keywords:** immune checkpoint inhibitors, immunotherapy, CTLA-4, PD-1, PD-L1

## Abstract

The discovery of immune checkpoint proteins such as PD-1/PDL-1 and CTLA-4 represents a significant breakthrough in the field of cancer immunotherapy. Therefore, humanized monoclonal antibodies, targeting these immune checkpoint proteins have been utilized successfully in patients with metastatic melanoma, renal cell carcinoma, head and neck cancers and non-small lung cancer. The US FDA has successfully approved three different categories of immune checkpoint inhibitors (ICIs) such as PD-1 inhibitors (Nivolumab, Pembrolizumab, and Cemiplimab), PDL-1 inhibitors (Atezolimumab, Durvalumab and Avelumab), and CTLA-4 inhibitor (Ipilimumab). Unfortunately, not all patients respond favourably to these drugs, highlighting the role of biomarkers such as Tumour mutation burden (TMB), PDL-1 expression, microbiome, hypoxia, interferon-γ, and ECM in predicting responses to ICIs-based immunotherapy. The current study aims to review the literature and updates on ICIs in cancer therapy.

## 1. Introduction

In recent years, considerable progresses have been achieved particularly in the field of personalised medicine, and cancer therapeutics [1]. Immunotherapy such as adoptive cell transfer (ACT), and immune checkpoint inhibitors (ICIs), is a type of cancer therapy, taking advantage of immune system components to fight tumour cells [2]. Immunotherapy alone or in combination with conventional treatments, such as radiotherapy and chemotherapy has achieved considerable success as a standard treatment in a number of cancers [3]. Programmed cell death 1 (PD-1) and cytotoxic T lymphocyte antigen 4 (CTLA-4) are co-inhibitory receptors expressed on the surface of T cells to negatively regulate T cell-mediated immune responses; however, tumour cells exploit these inhibitory molecules in order to induce tumour tolerance and T cell exhaustion [2]. Accordingly, ICIs such as anti-CTLA-4, anti-PD-1, and anti-PD-L1 can attach to these co-inhibitory receptors, thereby reactivating the immune response against tumour cells [4]. Three different groups of ICIs, including PD-1 inhibitors (Nivolumab, Pembrolizumab, and Cemiplimab), PDL-1 inhibitors (Atezolimumab, Durvalumab and Avelumab), and CTLA-4 inhibitor (Ipilimumab) have been approved by the US Food and Drug Administration (FDA) for the treatment of various types of cancer [5]. Nevertheless, only a subset (20–40%) of patients benefit from this therapy, highlighting the growing need to develop predictive biomarkers [6]. Tumour mutational burden (TMB), PDL-1 expression, microbiome, hypoxia, interferon-γ, extracellular matrix (ECM) and molecular and cellular characterization within the tumour microenvironment (TME) are all related to immunotherapy outcomes [2]. In addition, patients with advanced hepatocellular carcinoma (HCC) showed a poor overall survival (OS) to novel treatments, highlighting the role of biomarkers in predicting responses to ICIs [7]. Furthermore, it was reported that combination therapy of ICIs plus tyrosine kinase inhibitors (TKIs) in patients with metastatic renal cell carcinoma (mRCC) were more beneficial compared to sunitinib monotherapy in these patients [8]. Therefore, analysing the gene expression and spatial organization of the complex and heterogeneous tumours and their microenvironment could aid in the discovery of predictive biomarkers of response to immunotherapy [9,10,11,12]. The current study aims to study the recent advances in ICI-based immunotherapy and related biomarkers in predicting responses to immunotherapy.

## 2. Immune Checkpoint Inhibitors (ICIs)

ICIs are cancer immunotherapies that boost anti-cancer immune responses by targeting immunologic receptors on the surface of T-lymphocytes (Table 1) [13]. Therefore, ICIs were considered a novel treatment option in 2011 with the approval of ipilimumab [14], revolutionizing cancer treatment. These medications allowed for long-lasting results with a lower toxicity profile in some circumstances [15]. In contrast to traditional therapeutic strategies, ICIs work by reinvigorating the host immune system to fight tumour cells [16]. Immune checkpoints maintain a balance between pro-inflammatory and anti-inflammatory signals under homeostatic conditions [16]. These immunological checkpoints are a group of inhibitory and stimulatory pathways that influence immune cell activity [17]. Antibodies targeting immune inhibitory receptors, such as CTLA-4, PD-1, and PD-L have been the most widely used immunotherapeutic agents in the last decade [4]. Several antibodies and small compounds targeting various immune checkpoint proteins are in clinical development including B7H3, CD39, CD73, the adenosine A2A receptor, and CD47 [18].

Recent studies have identified several new immune checkpoint targets, such as lymphocyte activation gene-3 (LAG-3), T cell immunoglobulin and mucin-domain containing-3 (TIM-3), T cell immunoglobulin and ITIM domain (TIGIT), V-domain Ig suppressor of T cell activation (VISTA), and so on [29]. These experiments indicated that the blockade of a single immune checkpoint may lead to compensatory upregulation of other checkpoint receptors in TME [30]. The similar compensatory mechanism between TIM-3 and PD-1 was observed in lung cancer [31].

### 2.1. PD-1 Inhibitors

**Figure 1:**
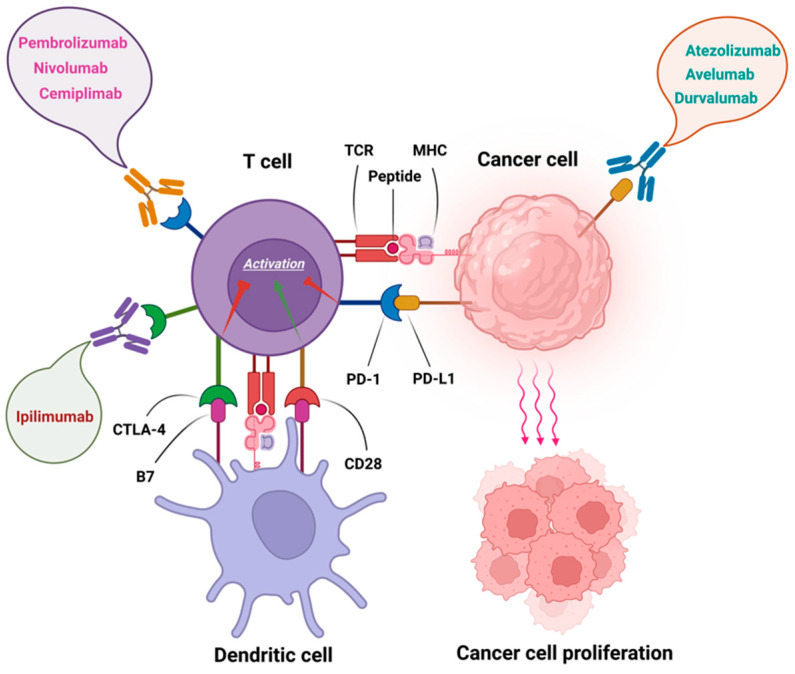
Immune checkpoint inhibitors approved by FDA. Pembrolizumab, Nivolumab, and Cemiplimab as anti-PD-1 antibodies, Ipilimumab as an anti-CTLA-4 antibody, as well as Atezolizumab, Avelumab, and Durvalumab as anti-PD-L1 antibodies.

PD-1 is an inhibitor receptor, playing a key role in programmed death signaling in order to regulate T- cell mediated responses [32]. PD-1 engagement can reduce cytokine secretion such as IL-2, IFN-γ, and TNF-α as well as cell proliferation through interfering with CD28-costimulatory signalling pathway [33]. The expression of PD-1 has been detected on various types of immune cell within TME, including activated monocytes, dendritic cells (DCs), natural killer (NK) cells, and T cells as well as B cells [33]. It has been demonstrated that immunotherapies, targeting PD-1 pathway, have revolutionized the treatment landscape of different cancers, including Merkel cell carcinoma (MCC), melanoma, head and neck squamous cell carcinoma (HNSCC), and non-small-cell lung cancer (NSCLC) [34]. The US FDA has approved three monoclonal antibodies namely Nivolumab, Pembrolizumab, and Cemiplimab as PD-1 inhibitors (Figure 1) [34].

Nivolumab (BMS-936558, ONO-4538, or MDX1106, trade name Opdivo; Bristol-Myers Squibb, Princeton, NJ, USA) is a first-in-class fully human immunoglobulin G4 (IgG4) monoclonal antibody (mAb) inhibitor that suppresses PD-1 activity through selectively targeting and blocking the interaction between ligands (PD-L1 and PD-L2) and PD-1 receptor [35]. Tumour cells have been shown to avoid immune surveillance by hijacking the PD-1/PD-L1 pathway, resulting in a reduced cellular immune response [35]. Nivolumab was approved by the FDA in 2014 and 2015 for the treatment of melanoma and renal cell carcinoma, respectively (Table 1). Furthermore, the FDA approved nivolumab in 2015 for the treatment of squamous cell lung cancer (SCLC) and NSCLC [36]. In 2010, Brahmer et al. demonstrated clinical activity of MDX-1106 in patients with various tumour types such as colorectal cancer, renal cell cancer, melanoma, NSCLC, and castration refractory prostate cancer [37]. Despite the presence of immune-related adverse events (irAE) such as renal, gastrointestinal, pulmonary, hepatic, cutaneous, rheumatological, and endocrine manifestations in patients with metastatic renal cell cancer, nivolumab proved to have a favorable safety profile [38].

Pembrolizumab (Keytruda, Merck) is another humanized IgG4 mAb that disrupts the PD-1/PD-L1 pathway [5]. Pembrolizumab has been approved by the FDA for the treatment of a variety of tumour types based on the observed robust objective responses and an excellent pharmacokinetic and safety profile [5]. The FDA recently confirmed (13 October 2021) that a combination of pembrolizumab and chemotherapy drugs, with or without bevacizumab, can have therapeutic benefits for patients with recurrent metastatic cervical cancer whose tumour cells express high levels of PD-L1 [39]. Several studies have found that pembrolizumab induces complete and robust responses in a tumour-agnostic manner, targeting the immune system rather than the tumour cell itself [40]. FDA recently approved the pembrolizumab as first tissue-agnostic/site-agnostic drug for treatment of patients with mismatch repair deficient/metastatic microsatellite instability—high (dMMR/MSI-H) [40]. FDA-approval of pembrolizumab introduces this mAb as a potential therapeutic agent for patients with advanced rare cancers; however, clinical evidence for the drug’s efficacy/safety profile in these patients needs to be investigated further [41]. As shown by Sundahl et al., pembrolizumab might enhance overall survival (OS) in patients suffering from metastatic urothelial carcinoma (UC) [42]. A phase II clinical trial (NCT02335424) of pembrolizumab on 370 patients with UC reported a satisfactory durable response rate (DRR) in cisplatin-ineligible patients [43]. It also demonstrated considerable anticancer efficacy in HNSCC, as shown by enhanced overall response rate ORR. A phase III study (KEYNOTE-048) date demonstrated that Pembrolizumab compared to chemotherapy has remarkably improved OS in recurrent/metastatic (R/M) HNSCC patients with PD-L1 combined positive score (CPS) ≥20 and its combinations with chemotherapy was more efficacious compared to chemotherapy in R/M HNSCC patents with PD-L1 CPS ≥1, proposing c [44]. A study (NCT02358031) reported a successful combination of pembrolizumab with platinum and 5-FU, highlighting their potential therapeutic option as a first-line treatment in patients with HNSCC [45].

Cemiplimab (Libtayo^®^, Regeneron Pharmaceuticals/Sanofi) is considered a fully humanized IgG4 mAb that inhibits the interaction of the PD-1 receptor with its ligands and is used to treat patients with metastatic or locally advanced cutaneous squamous cell carcinoma (CSCC) who are ineligible for curative resection or radiotherapy (approved by FDA in September 2018, and by the European Medicines Agency (EMA) in June 2019) [46]. Cemiplimab was the first drug approved by the FDA for the treatment of CSCC, and it is also mentioned and recommended in the 2020 European interdisciplinary guidelines (issued by the EDF, EADO, and EORTC) as the first-line therapy for cancer patients who cannot be treated with radiotherapy or surgery [47]. Furthermore, the UK National Institute of Health and Care Excellence (NICE) and National Comprehensive Cancer Network (NCCN) have recommended Cemiplimab as a treatment option for treating locally advanced or metastatic CSCC in patients who are not candidates for curative resection or radiation therapy [48]. In patients with metastatic CSCC, Cemiplimab has exhibited remarkable anticancer activity with a reasonable safety profile [49]. Fatigue (27.0 percent) and diarrhea (23.5 percent) are the most prevalent side effects reported in patients with CSCC during or after therapy with Cemiplimab (3 mg/kg) [50]. Cemiplimab was also linked to improved OS and PFS in CSCC patients in comparison to EGFR inhibitors and chemotherapy, showing that it has a lot of potential in treating CSCC patients [50]. It may result in better OS and PFS compared to platinum-based chemotherapy in patients with advanced NSCLC, indicating a possible new treatment option for these patients [51].

### 2.2. PD-L1 Inhibitors 

PD-1 ligand 1 (PD-L1) and PD-L2 are the two ligands for PD-1 [52]. Both tumour and immune cells can express PD-L1 which is a useful biomarker in predicting response to anti-PD-1/PD-L1 antibodies in some patients with different types of cancer [53]. PD-L1, also known as B7-H1 or CD274, plays a part in inhibiting cancer-immunity cycle through binding to negative regulators of T-cell activation such as PD-1 and B7.1 (CD80) [54]. Therefore, PD-L1 ligation is known to inhibit migration and proliferation of T cell, thereby restricting tumour cell killing (Figure 1) [55]. The US FDA has approved three PD-L1 inhibitors namely Atezolimumab, Durvalumab and Avelumab that have been used in some solid tumours, including NSCLC, HNSCC, melanoma, and MCC [34].

Atezolizumab (MPDL3280, Tecentriq, Genentech, Inc., San Francisco, CA, USA) is a IgG1 monoclonal antibody, designing with Fc domain modification in order to reduce antibody-mediated cellular cytotoxicity, thereby preventing T cell depletion expressing PD-L [56]. It has been approved by the FDA for adjuvant therapy following surgery and chemotherapies in patients with stage II and IIIA NSCLC whose tumours have PD-L1 expression on ≥1% of tumour cells in October 2021 [57]. Furthermore, based on the Phase 3 IMpassion130 trial, the FDA granted atezolizumab accelerated approval in March 2019 [58]. These trial results demonstrated that Atezolizumab improved progression-free survival in patients with metastatic breast cancer. Atezolizumab inhibits PD-L1 to lower immunosuppressive signals in TME, thereby increasing T cell-mediated immunity against malignancies [59]. In 2016, the FDA approved Atezolizumab for progressed metastatic NSCLC patients, receiving platinum-containing chemotherapy [60]. Patients with recurrent NSCLC tumours, expressing medium or high levels of PD-L1, Atezolizumab demonstrated a statistically significant survival advantage in comparison with docetaxel (HR = 0.54; *p* = 0.014) in a randomized phase II trial (POPLAR) [61]. A study evaluated the effectiveness and safety of nab-paclitaxel with or without Atezolizumab in 451 patients with treatment-naive metastatic triple negative breast cancer (TNBC) until progression of the disease. A median follow-up taking up to 12.9 months revealed that the Atezolizumab to Nab-paclitaxel combination decreased by 40% the risk of progression or death in patients PD-L1-positive tumours in comparison with nab-paclitaxel alone [62]. 

Avelumab (MSB0010718C) is another completely human IgG1 monoclonal antibody, binding to PD-L1, thereby inhibiting PD-L1 and PD-1 interactions. This could lead to T cells mediated antitumour responses as well as [63]. Based on the evidence from Part A of the JAVELIN Merkel 200 clinical trial, avelumab was designated as a breakthrough therapy by the FDA in November 2015 for treating patients with metastatic MCC who had disease progression after previous chemotherapy [64]. In March 2017, avelumab was finally designated as a breakthrough therapy and was approved by the FDA for patients with metastatic MCC, regardless of previous chemotherapy [63]. 

A three-year follow-up of a trial investigating long-term safety revelated no adverse events in patients with MCC following Avelumab administration, highlighting avelumab’s efficacy as a SOC therapy for these patients [65]. Furthermore, Avelumab, in combination with Axitinib, is currently regarded as first-line therapy for patients with advanced renal cell carcinoma (RCC) [66,67]. In contract to Sunitinib, an FDA-approved receptor tyrosine kinase inhibitor, the addition of avelumab to Axitinib could enhanced progression free survival (PFS) in these patients [67]. 

Durvalumab (MEDI4736) is an FDA-approved immunotherapy, binding to PD-L1 with high affinity and specificity, thereby inhibiting its interactions to PD-1 and CD80 [68,69]. It was designated by the FDA as a breakthrough therapy in February 2016 based on early clinical data from a Phase I trial for treating patients with metastatic urothelial bladder cancer whose tumour cells express PD-L1 and who had advanced disease during or after one standard platinum-containing chemotherapy regimen [70]. Durvalumab was also approved by the FDA in May 2017 for the treatment of patients with urothelial carcinoma (metastatic or locally advanced) who progressed during or after platinum-based chemotherapy, including those who had disease progression within one year of treatment with a platinum-based regimen in the neoadjuvant or adjuvant setting followed by surgical resection [70]. In 2018, FDA also approved this drug for patients with unresectable stage III NSCLC that their disease has not progressed after concomitant platinum-based chemotherapy and radiotherapy based on PACIFIC trial results [71]. It was thought that using it in combination with chemotherapy, immunotherapy, and targeted treatment would optimize benefit. However, patients with PD-L1 ≥25%, receiving Durvalumab, had numerically longer median OS (16.3 months) compared with those received chemotherapy (12.9 months), whereas patients treated with Durvalumab/Tremelimumab combination had the median OS of 11.9 months, which was less than Durvalumab/chemotherapy combination, suggesting Durvalumab as an appropriate option for NSCLC patients [72].

### 2.3. CTLA-4 Inhibitor

CTLA-4 was found as a protein belonging to the immunoglobulin superfamily that was expressed primarily by activated T cells in a cytotoxic T lymphocyte cDNA library [73]. CTLA-4 is expressed solely on T cells and governs the amplitude of T cell activation throughout the early phases. CTLA-4 primarily inhibits the function of CD28, a T-cell co-stimulatory receptor [74]. Despite the fact that CTLA-4 binds to the same ligand B7 on B cells and APCs as its homologue CD28, stimulation of CTLA-4 resulted to T cell-mediated suppression of antibody formation and avoidance of allograft rejection [75,76]. CTLA-4 expression kinetics were discovered to be substantially different from CD28 expression in 1994. CTLA-4 expression is increased for 2–3 days after TCR/CD3-mediated T cell activation, commencing about 24 h after TCR triggering, whereas CD28 is expressed on naive T cells. These findings suggest that CTLA-4 is critical in the regulation of activated T cells, as the absence of CTLA-4 results in unregulated T cell proliferation. Because of these new insights into CTLA-4’s mode of action, researchers decided to see if blocking CTLA-4 may increase antitumour immune responses [77].

CTLA-4 inhibition improves a wide range of immunological responses that rely on helper T cells, whereas CTLA-4 interaction on T_reg_ cells improves their suppresisive activity. T_reg_ cells produce CTLA-4 constitutively because it is a target gene of the forkhead transcription factor FOXP3 [78], whose expression determines the T_reg_ cell lineage [79]. T_reg_ cell-specific CTLA-4 deletion or inhibition greatly decreases their ability to control both autoimmune and antitumour immunity, despite the fact that the mechanism by which CTLA-4 promotes the immunosuppressive activity of Treg cells remains unknown [80]. As a result, both increase in effector CD4+ T cell activity and reduction in Treg cell-dependent immunosuppression are likely essential aspects in CTLA-4 blockade’s mode of action.

Ipilimumab (Yervoy) is a human IgG1 mAb that can inhibit the function of CTLA-4 and was first approved and recommended for the treatment of melanoma in 2011 [81]. Ipilimumab is also used in the treatment of advanced renal cell carcinoma, MSI-H/dMMR metastatic colorectal cancer, malignant pleural mesothelioma, NSCLC, and hepatocellular carcinoma when combined with Opdivo (nivolumab) [82]. In 2020, the FDA announced that Opdivo (nivolumab) plus Yervoy (ipilimumab) (given as intravenous injections) have therapeutic benefits for adult patients with unresectable malignant pleural mesothelioma (MPM), and NSCLC (with tumour PD-L1 expression ≥1% and no EGFR/ALK aberrations) as a first-line treatment [83]. Nevertheless, CTLA-4 as a negative regulator of T cell immunological responses, is implicated in autoimmunity prevention; therefore, its blockage with ipilimumab may cause immune related adverse effects (irAEs) such as colitis and enterocolitis [84].

Recently, ICI therapy has become a promising therapeutic strategy with encouraging therapeutic outcomes due to their durable anti-tumour effects. Though, tumour inherent or acquired resistance to ICIs accompanied with treatment-related toxicities hamper their clinical utility [85]. Accordingly, several studies have exhibited that combination therapy with ICIs plus other therapeutic approaches, such as chemotherapy [86], radiotherapy, cancer vaccines, and also CXCR4 blockade therapy [87] can efficiently circumvent tumour resistance to ICI therapy.

Researchers have found that the combination of cyclophosphamide, ICI, and vinorelbine inhibits TNBC growth mainly by inducing APC recruitment and also activation in vivo [88]. Additionally, CTLA-4 inhibitors monotherapy as well as combination therapy with CTLA-4 inhibitors and either gemcitabine or cyclophosphamide showed promising results in BC and also CRC mouse models [89]. The phase 1 clinical trial of 15 patients with refractory and metastatic HNSCC showed that combination therapy of cyclophosphamide and radiation therapy in combination with GM-CSF (granulocyte macrophage-colony-stimulating factor) could demonstrate significant therapeutic benefit [90]. Recent research demonstrated that treatment with PD-L1 and CTLA-4 inhibitors in conjunction with cancer stem cell-pulsed dendritic cells (CSC-DC) improved T cell proliferation, inhibited TGF-β secretion, intensified IFN-γ secretion, and improved host-specific CD8^+^T cell response versus CSCs in B16-F10 mice melanoma tumour model [91]. In melanoma, prostate, and also PDA murine model, GMCSF cell-based vaccines combined with CTLA-4 inhibitor decreased tumour growth and restored the antitumor immunity [92,93,94]. The combination of RT with targeting CTLA-4 and/or PD-1/PD-L1 has been shown to trigger CTL-mediated antitumor immunity [95]. For instance, in glioma xenograft-bearing mice, combining PD-1 blockade and dose brain-directed radiation (10 Gy) glioma xenograft-bearing mice resulted in a 75% complete pathologic response, as well as improving OS largely due to activation of CTLs and macrophages [96]. 

Drugs blocking these pathways are currently utilized for a wide variety of malignancies and have demonstrated durable clinical activities in a subset of cancer patients. New inhibitory pathways are under investigation, and drugs blocking LAG-3, TIM-3, TIGIT, VISTA, or B7/H3 are being investigated. Given its unique mechanism of action compared to other anticancer strategies, next generation of immune checkpoints appears to have a synergistic effect when combined with chemotherapy or other ICIs [97].

## 3. Biomarkers for ICI-Based Immunotherapy

Various biomarkers such as PD-L1 expression, tumour mutation burden (TMB), microsatellite instability, microbiome, hypoxia, interferon-gamma (IFN-γ), and extracellular matrix have been reported on order to increase response to immunotherapy in patients, receiving ICIs. 

### 3.1. PD-L1 Expression

PD-1 is a signalling receptor expressed on the surface of T lymphocytes [98]. PD-1 and its ligand programmed cell death protein-1 ligand (PD-L1) have been studied broadly in clinical trials as biomarkers for ICI based immunotherapy [99,100]. Expression of PD-L1 was found to be increased by inflammatory factors particularly, interferon-γ in TME [101]. The expression of PD-L1 was also found to impair cytotoxic T lymphocyte (CTL) protection and reduce chronic viral infections [100]. The PD-L1 expression is scored by pathologists and is defined with immunohistochemistry (IHC) [102]. To reinvigorate the immune system to fight tumour cells, the PD-1/PD-L1 interaction could be considered a potential target for some monoclonal antibodies (mAbs), resulting in the inhibition of this interaction [103]. In a study conducted by Bellmunt et al., 542 patients with advanced urothelial cancer were evaluated, and it was found that Pembrolizumab had an improved survival and fewer side effects than chemotherapy [104]. KEYNOTE-522 compared patients who received Pembrolizumab and chemotherapy to patients who received placebo and chemotherapy and found that the first group had better pathological outcomes [105]. KEYNOTE-024 trail revealed that a fixed dose of Pembrolizumab (200 mg) was associated with the improved OS and PFS and lower treatment-related adverse events in NSCLC patients with PD-L1 tumour proportion score (TPS) ≥50% in comparison with chemotherapy [86]. Furthermore, KEYNOTE-048 results showed that Pembrolizumab improved OS in patients with R/M HNSCC as PD-L1 increases, demonstrating the role of the PD-L1 expression is a demonstrator of response for ICIs [44]. According to the results of CHECKMATE 040, combination therapy of Nivolumab plus Ipilimumab has been approved as second-line therapy for hepatocellular carcinoma (HCC) in patients receiving sorafenib [106]. 

### 3.2. Tumour Mutation Burden

TMB has been defined as the number of mutations in cancer cells’ DNA, which is reported as mutations per megabase (mut/Mb) [107]. TMB is determined using either NGS or PCR, and it may aid in the selection of the best ICIs [108]. In patients with NSCLC, it was reported that if TMB was equal to or greater than 10 mut/Mb, the combination of Nivolumab and Ipilimumab would have a better overall response (OR) regardless of PD-L1 expression [109]. TMB ≥ 10 mut/Mb is considered as High TMB (TMB-H), and it has been linked to improved survival in a variety of tumour types, including small cell lung cancer (SCLC) [110]. KEYNOTE-158 discovered that Pembrolizumab, as an ICI, is effective in various types of cancers with TMB ≥10 mut/Mb, particularly solid cancers [111]. Yefarchoan and colleagues evaluated the correlation between TMB and the objective response rate (ORR) in 27 tumour types among patients, receiving anti-PD-1 or anti-PDL-1 therapy, and observed that some cancers such as MCC responded to therapy better than what was predicted by TMB, suggesting that the emergence of viral antigens on some tumours may increase the response to anti-PD-1 therapy [112].

### 3.3. Microsatellite Instability (MSI)/DNA Mismatch Repair (dMMR)

Generally, DNA mismatch repair system (MMR) is a pivotal system to recognize and repair base-base mismatches and misincorporation errors occurred during DNA replication [106]. Therefore, any deficiency in this system could lead to hypermutation in cancer [113]. Microsatellite instability (MSI) status has been evaluating as a potential predicting biomarker in cancer immunotherapy [113]. Currently, discovered that MSI/dMMR could be a prognostic test in ICI-based immunotherapy to show how tumours respond to antibodies, particularly in cancers such as colorectal, gastric cancer and endometriosis [114]. IHC and PCR are of great importance to detect mismatch repair proteins such as MSH2/6, MLH1, and PMS2 and also small genomic alterations, respectively [115]. Microsatellite unstable tumours have been categorized into two phenotypes namely MSI-low (MSI-L) and MSI-high (MSI-H) that the latter enhanced significantly the neoantigen load, leading to activating lymphocyte, thereby rendering tumour cells sensitive to ICIs [116]. It was reported that its combination with TMB and PD-1/PD-L1 expression could play an important role in predicting responses to immunotherapy [116]. The CHECKMATE 142 study reported that Nivolumab could provide a durable response in patients with dMMR/MSI-H metastatic colorectal cancer, proposing Nivolumab as a new treatment option in these patients [117]. 

### 3.4. Microbiome

Microbiota in the human body can be found in all parts of the body, but particularly in the skin, saliva, and gastrointestinal tract [118]. The microbiome can influence ICI therapy by influencing the immune system [119]. An in vivo study using CT26 tumour-bearing mice revealed that high microbial diversity could affect ICI response efficiently by increasing IL-2 and IFN- secretion when compared to mice given antibiotic injection [120]. As a result, it has been proposed that the microbiome can enhance immune response, stimulate inflammation, or disrupt the balance of proliferation and cell death, thereby increasing tumourigenesis [120]. It has been reported that the gut microbiome activates T cell-mediated responses, resulting in the direct targeting of tumour cells [121]. Furthermore, melanoma patients treated with anti-PD-L1 had higher levels of Bifdobacterium longum, Collinsella aerofaciens, and Enterococcus faecium, highlighting the importance of the microbiome [122]. In recently published work, Zheng et al. reported some dynamic alterations within gut microbiome in patients with HCC during their immunotherapy. Moreover, metagenomic sequencing data showed higher taxa in faecal samples of patients, responding to immunotherapy compared to non-responders, highlighting the role of microbiome in regulating immune responses [123].

### 3.5. Hypoxia

Hypoxia is measured using specialized techniques such as PO2 electrode measurement, fibre optic probe, and nuclear magnetic resonance (NMR) [124,125]. A lack of oxygen causes hypoxia-inducible factor-1 (HIF-1), a transcription factor, to be activated within the TME, and its subunit can promote angiogenesis and metastasis [126,127]. Hypoxia has been shown to modulate the expression of immune checkpoints such as CTLA-4, CD47, PD-1/L-1, and TIM3 in order to manipulate immune cells’ mediated anti-tumour response, thereby inhibiting immune surveillance [126]. In hypoxic conditions, the combination of adenosine and the A2a receptor increases immune checkpoint expression, resulting in T cell suppression [126]. Zandberg et al. reported that in an animal model of HNSCC, disease control rate (DCR) and survival were altered in mice with intra-tumoral hypoxia, rendering them resistant to anti-PD-1 immunotherapy [128]. Furthermore, the study 111/KEYNOTE-146 trial (NCT02501096) showed that Pembrolizumab plus lenvatinib, a VEGFR1/2/3 kinases inhibitor, resulted in a promising antitumour response as well as a manageable safety profile in patients with metastatic renal cell carcinoma (RCC) who have been previously treat with ICI therapy [129]. 

### 3.6. Interferon-γ 

Different immune cells within the TME, such as lymphocytes and NK cells, can produce a variety of inflammatory mediators and cytokines [130]. Interferon- (IFN-) is a type II interferon that has both antitumour and proliferative effects [131]. Although CD8^+^ cytotoxic T lymphocytes (CTLs) are the primary producers of IFN- γ, IL12, IL15, and IL18, as well as pathogen-associated molecular patterns (PAMPs), have been shown to stimulate IFN-γ secretion [132,133,134]. The main IFN-γ signalling pathway is JAK/STAT, which regulates immune responses and tumourigenesis [131,132]. IFN-γ promotes chemotaxis and immune cell recruitment to TME by inducing the transcription and production of C-X-C motif chemokines (CXCL) 9, 10, and 11, as well as their receptor CXCR3 [133]. However, IFN-γ can promote tumourigenesis by increasing the expression of indoleamine 2,3dioxygenase 1 (IDO1), CTLA-4, and PD-L1 [135,136]. IDO1 is an enzyme that reduces CD8 cell activity by converting tryptophan to kynurenines, emphasising its immunosuppressive role [137]. Given this information, the IFN-γ signature, which includes CXCL10, CXCL9, HLA-DRA, IDO1, STAT1, and IFNG, is significant in predicting responses to ICIs therapy [131]. 

### 3.7. Extracellular Matrix 

Extracellular matrix (ECM) is a three-dimensional network composed of extracellular macromolecules that provides biochemical support to tissue [138,139]. Desmoplasia, or connective tissue growth, has been linked to a poor prognosis in patients with solid tumours due to high collagen and fibroblast infiltration within TME [140]. This can result in stiffness, which is the primary distinction between normal and cancerous ECM [141]. Metalloproteases (MMPs) can break down ECM ingredients to generate some fragments of macromolecules, showing either pro-or anti tumourigenic functions in different types of cancers [142]. As a result, collagen IV-derived fragments such as tetrastatin, canstatin, and tumstatin can reduce the invasiveness and proliferative properties of tumour cells by binding to integrins (α3β1, α5β1, or αVβ3) [142,143]. Furthermore, Lysyl oxidase (LOX) inhibits T cell migration to ECM and suppresses immune response. The KPC model was used to demonstrate that inhibiting LOX can increase T cell infiltration, thereby improving responses to ICI-based immunotherapy [142,143]. 

## 4. Conclusions and Future Perspectives

ICI treatment has completely transformed cancer immunotherapy. We looked at the history and development of the most well-known ICIs, as well as the issues they pose in the clinic, such as treatment resistance and adverse events (irAEs). While ICIs are the most often utilized, they are not the only FDA-approved immunotherapies. The FDA’s approval of anti-CTLA-4 therapy, followed by reports of promising preliminary clinical results for anti-PD-1 therapy, has sparked a renewed interest among oncologists in the endogenous immune system’s potential antitumour activity after the immune system’s ‘brakes’ have been released. The discovery of immune checkpoints such as CTLA-4 and PD-1 has unquestionably aided the advancement of cancer immunotherapy. Although these molecules were initially identified to serve a function in T cell activation or death, additional preclinical studies revealed that they also play a crucial role in the maintenance of peripheral immunological tolerance.

Depending on the preclinical model, blocking CTLA-4 and PD-1 resulted in the formation of anticancer immune responses that were successful as single agents or required further therapy [4]. As a result, it is remarkable that single-agent anti-CTLA-4 and anti-PD-1 anticancer therapies are so successful. Immune checkpoint drugs have ushered in a new era in metastatic cancer treatment. Other immune checkpoints or inhibitory receptors have been revealed that can be targeted by monoclonal antibodies based on their cell surface expression, in addition to CTLA-4 and the PD-1/PD-L1 axis. Inhibitory receptors such as TIM-3, LAG-3, and BTLA are examples of inhibitory receptors, some of which appear to have a function in immunological tolerance while others appear to play a far more modest role [144]. 

As a result, in addition to examining the function of existing molecules in cancers other than melanoma, NSCLC, and RCC, novel combinations will be tested in early clinical trials. Furthermore, depending on clinical responses in diverse cancer types, checkpoint inhibitors, as single medicines or in combination, will likely be investigated in adjuvant or neoadjuvant methods, with the goal of improving the overall survival of many cancer patients. These treatments have revolutionized cancer immunotherapy by demonstrating, for the first time in many years of research, an improvement in overall survival in metastatic melanoma, one of the most immunogenic human cancers, with an increasing number of patients benefiting long-term from these treatments. However, the function of systemic immunity in these modalities is not fully known. Researchers must also look at the systemic effects of various immunotherapies in order to gain a better knowledge of how the immune system begins and maintains an effective antitumour response. Taken together, much work has been carried out and more is required to uncover the underlying mechanisms in response to ICIs in patients with various types of tumours. It seems that combination therapy with other ICIs, chemotherapy, targeted agents, radiotherapy, and T- cell based therapies could improve ICI outcomes, particularly in patients who have not experienced a favourable response to ICI-based therapies. 

Even though ICIs have shown promising results in adults, there is limited data regarding their safety in children [145]. It was demonstrated that dose dependent adverse events of CTLA-4 blockade ranging from mild to moderate occurred in more than 70% of patients [146]. Furthermore, a meta-analysis of 18 clinical trials revealed an increased risk of treatment-related mortality (TRM) in patients receiving higher dose (10 mg/kg) of CTLA-4 inhibitors [146]. The toxicities corelated with PD-1/PD-L1 blockades were less severe compared to CTLA-4 inhibitors, and fatigue as the most common adverse event, occurring in 16–37% of patients receiving PD-1 inhibitor, and 12–24% of those who received PD-L1 inhibitor [147]. It is particularly concerning that unpredictable off-target effects on critical organs can pose a life-threatening risk to children whose organs are less mature, thereby presenting a potential life-long disability danger [147]. Moreover, TME as a major composition of cancer and immune cells is of great importance that may complicate the treatment process. 

## Figures and Tables

**Table 1:** The list of ICIs with the cancer type indication.

Drug	Target	Approval	FDA-Approved Indications	References
Nivolumab	PD-1	March 2015	Stage III-B or IV Squamous NSCLC	[19]
Pembrolizumab	PD-1	October 2016	Stage IV nonsquamous and squamous NSCLC	[20]
Atezolizumab	PD-L1	October 2016	Stage III-B or IV nonsquamous and squamous NSCLC	[21]
Cemiplimab	PD-1	September 2018	metastatic cutaneous squamous cell carcinoma	[22]
Ipilimumab	CTLA-4	August 2010	stage 3 or 4 malignant melanoma	[23]
Avelumab	PD-L1	March 2017	histologically confirmed metastatic Merkel cell carcinoma	[24]
Durvalumab	PD-L1	February 2016	Stage III non-small-cell lung cancer (NSCLC)	[25]
Pembrolizumab + cis/carboplatin + pemetrexed	-	August 2018	Nonsquamous NSCLC	[26]
Pembrolizumab + paclitaxel/nab-paclitaxel + carboplatin	-	October 2018	Stage IV Squamous NSCLC	[27]
Atezolizumab + carboplatin + paclitaxel + bevacizumab	-	December 2018	Stage IV NSCLC	[28]
